# Staged open surgery for bicuspid aortic valve regurgitation and coarctation of the aorta in a Jehovah’s witness

**DOI:** 10.1186/s12872-020-01507-z

**Published:** 2020-05-11

**Authors:** Kohei Sumi, Shigehiko Yoshida, Yoshitaka Okamura, Tomokazu Nakamura

**Affiliations:** 1 Department of Cardiovascular Surgery, IMS Tokyo Katsushika General Hospital, 4-18-1, Nishishinkoiwa, Katsushika-ku, Tokyo, 124-0025 Japan; 2 Department of Cardiovascular Surgery, Seiyu Memorial Hospital, Wakayama, Japan

**Keywords:** Coarctation of the aorta, Bicuspid valve, Jehovah’s witness

## Abstract

**Background:**

Jehovah’s Witnesses refuse allogeneic blood transfusions, which makes cardiovascular surgery challenging. Surgeons must minimize blood and fluid loss within one procedure.

**Case presentation:**

We herein describe a 17-year-old male Jehovah’s Witness with bicuspid aortic valve regurgitation and coarctation of the aorta. The procedures were performed in the following order: aortic valve replacement combined with Nick’s aortic root enlargement, right axillary artery–bilateral external iliac artery bypass, and distal arch–descending aorta bypass.

**Conclusions:**

Axillary artery–bilateral external iliac artery bypass maintained distal perfusion and reduced the amount of heparin during distal arch–descending aorta bypass surgery.

## Background

Jehovah’s Witnesses refuse allogeneic blood transfusions. Thus, most centers do not offer cardiovascular surgery because of the high risk of bleeding-related mortality and morbidity. We herein describe a successful staged procedure for bicuspid aortic valve regurgitation and coarctation of the aorta.

## Case presentation

**Fig. 1:**
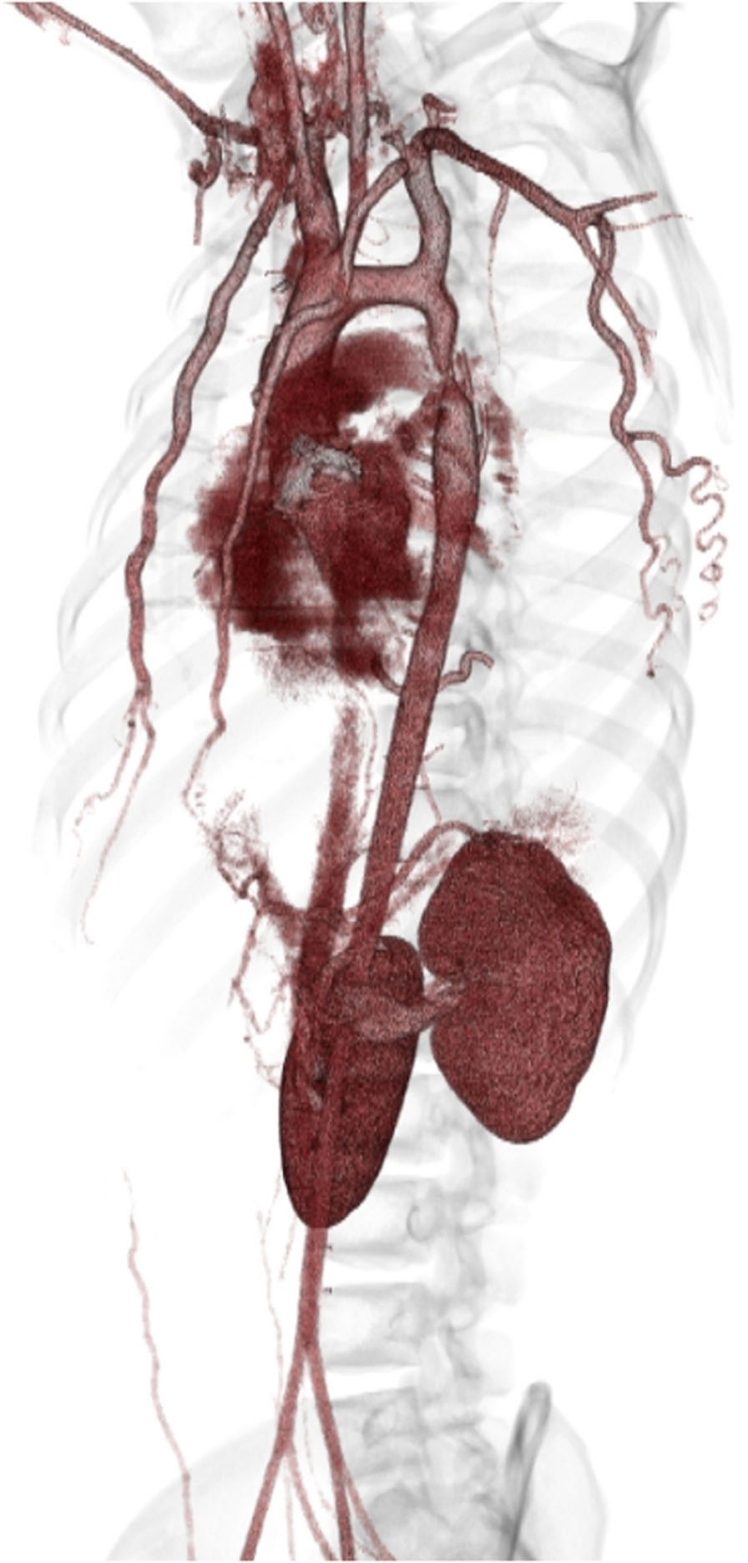
Computed tomography angiography showed coarctation of the aorta

A 17-year-old, 78-kg male Jehovah’s Witness was referred to our center for treatment of bicuspid severe aortic valve regurgitation and coarctation of the aorta. The patient had a history of intermittent claudication and hypertension (blood pressure, 160/65 mmHg). His ankle-brachial index was 0.59 and 0.54 on the right and left, respectively. Ultrasound examination revealed severe aortic regurgitation caused by a bicuspid valve. The annular size was 22 mm. Computed tomography angiography showed coarctation characterized by a narrowing immediately distal to the aortic isthmus (Fig. 1). Ascending aorta dilation was not observed (28 mm). The diameter of the distal arch and descending aorta was 14 mm and 20 mm, respectively. The patient refused transfusion of whole blood, packed red blood cells, platelets, or plasma regardless of his circumstances. He consented to undergo intraoperative cell salvage as long as continuity was maintained with the vascular system.

## Procedure

**Fig. 2:**
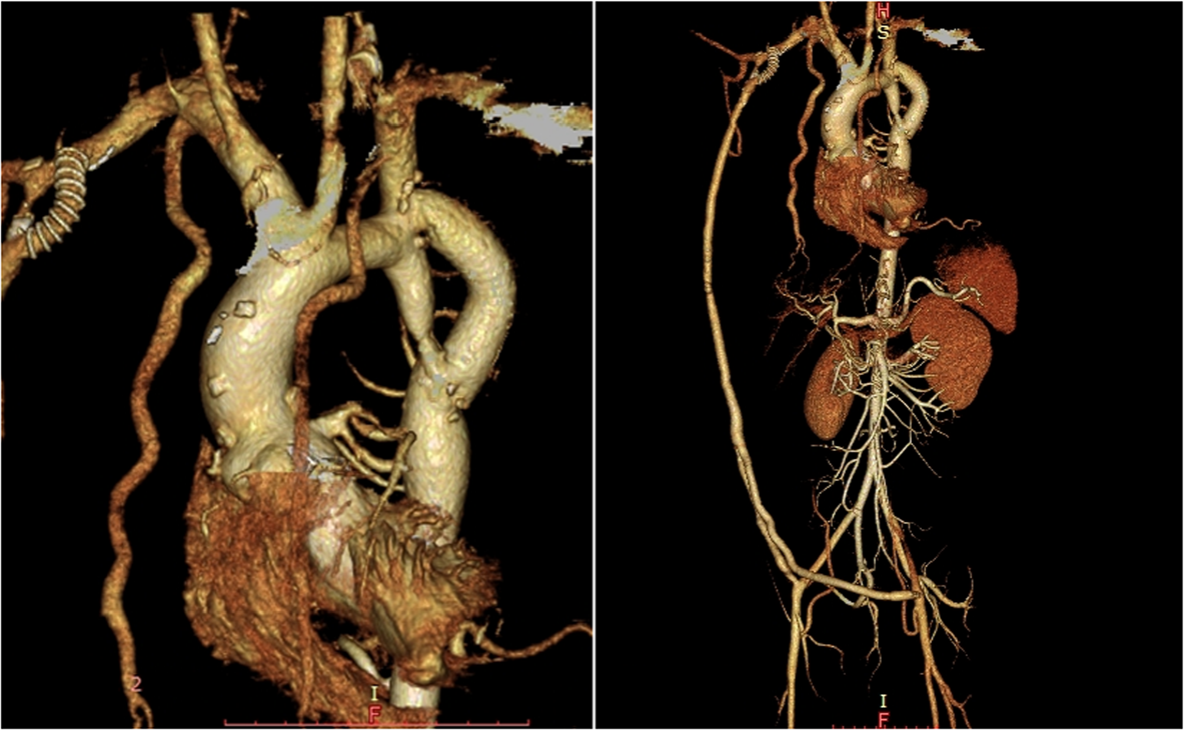
Postoperative multislice computed tomography angiography showed a patent graft

In the first stage (17-year-old), aortic valve replacement combined with Nick’s aortic root enlargement was performed with median sternotomy. Four months later, right axillary artery–bilateral external iliac artery bypass was performed. Four years later (21-year-old), axillary artery to prosthetic graft shunting was required for proximal anastomotic stenosis. The hypertension and intermittent claudication were relieved, but the symptoms gradually relapsed and his renal function worsened because of insufficient blood supply to the renal artery. Another approach to repair the coarctation was required. Six years later (23-year-old), we performed distal arch–descending aorta bypass by left lateral thoracotomy through the fourth intercostal space. After heparinization, we clamped the descending aorta and performed occlusion test for safety of simple clamping. Distal perfusion using cardiopulmonary bypass was not required because distal perfusion was maintained by the axillary–iliac artery bypass. A clamp was placed across the aortic arch immediately distal to the left common carotid artery, a second clamp was placed across the left subclavian artery, and a third was placed across the descending thoracic aorta immediately distal to the pathology. After longitudinal aortotomy, a 16-mm knitted Dacron tube prosthesis was anastomosed proximally by end-to-side anastomosis. The distal anastomosis was performed on the descending aorta immediately distal to the pathology (Fig. 2). The hemoglobin level preoperatively, postoperatively, and at discharge (9 days postoperatively) was 12.9, 11.6, and 12.4 g/dl, respectively. The serum creatinine level was 1.53 mg/dl preoperatively and 0.74 mg/dl postoperatively. The patient showed no paraplegia, and his ankle-brachial index increased to 0.88 and 0.93 on the right and left, respectively.

## Discussion and conclusion

Coarctation of the aorta and bicuspid aortic valve pathologies often coexist. The European Society of Cardiology 2014 guidelines recommend repairing coarctation of the aorta in patients with a noninvasive pressure difference of > 20 mmHg between the upper and lower limbs, regardless of symptoms but with upper limb hypertension (> 140/90 mmHg in adults) [1]. Stenting is now commonly performed in many centers [2], but it does not eliminate the risk of aortic rupture or dissection. Furthermore, stenting for coarctation of the aorta is not covered by insurance in Japan. In Jehovah’s Witnesses, staged procedures are favored for management of coarctation and cardiac abnormalities because blood and fluid loss during a single procedure must be minimized. Numerous operative techniques are available to manage the coarctation segment: resection of the stenotic segment with end-to-end anastomosis, aortoplasty using a prosthetic patch or the left subclavian artery, interposition grafting, and ascending or distal arch to descending aorta bypass. These procedures have potential complications including bleeding from collateral networks and paralysis due to spinal cord ischemia. Axillary artery–iliac artery bypass can reduce left ventricular afterload and increase distal perfusion, but it is not a fundamental solution because the bypass does not guarantee sufficient blood flow and patency. Comerota and White [3] described maintenance of distal perfusion using a temporary axillary–femoral artery bypass graft to reduce morbidity of thoracoabdominal aneurysm repair. This method does not require additional cardiopulmonary bypass and reduces the total amount of heparin. Cardiovascular surgery in Jehovah’s Witnesses is challenging. We have herein reported a case of a successful staged procedure for bicuspid aortic valve regurgitation and coarctation of the aorta.

## Data Availability

Not applicable.
